# Construction of plasmid-free *Escherichia coli* for the production of arabitol-free xylitol from corncob hemicellulosic hydrolysate

**DOI:** 10.1038/srep26567

**Published:** 2016-05-26

**Authors:** Buli Su, Zhe Zhang, Mianbin Wu, Jianping Lin, Lirong Yang

**Affiliations:** 1 Key Laboratory of Biomass Chemical Engineering of Ministry of Education, College of Chemical and Biological Engineering, Zhejiang University, Hangzhou 310027, China

## Abstract

High costs and low production efficiency are a serious constraint to bio-based xylitol production. For industrial-scale production of xylitol, a plasmid-free *Escherichia coli* for arabitol-free xylitol production from corncob hemicellulosic hydrolysate has been constructed. Instead of being plasmid and inducer dependent, this strain relied on multiple-copy integration of xylose reductase (XR) genes into the chromosome, where their expression was controlled by the constitutive promoter P43. In addition, to minimize the flux from L-arabinose to arabitol, two strategies including low XR total activity and high selectivity of XR has been adopted. Arabitol was significantly decreased using plasmid-free strain which had lower XR total activity and an eight point-mutations of XR with a 27-fold lower enzyme activity toward L-arabinose was achieved. The plasmid-free strain in conjunction with this mutant XR can completely eliminate arabitol formation in xylitol production. In fed-batch fermentation, this plasmid-free strain produced 143.8 g L^−1^ xylitol at 1.84 g L^−1^ h^−1^ from corncob hemicellulosic hydrolysate. From these results, we conclude that this route by plasmid-free *E. coli* has potential to become a commercially viable process for xylitol production.

In recent decades, many biotechnological approaches for xylitol production have been explored with the goal to develop an alternative process for superseding chemical hydrogenation^1^. However, the economics of bio-based xylitol production remain elusive by many reasons such as high costs and low production efficiency. In previous work, we have constructed a genetically modified *E. coli* for xylitol production from corncob hemicellulosic hydrolysate^2^. Yet the plasmid-based expression system used in the aforementioned work is limited by the metabolic burden on the host strain and the antibiotic resistance gene used in the plasmid. A general disadvantage of using antibiotic selection is the need to completely eliminate residual antibiotic from the final product before its application^3^. Furthermore, the cost associated with using antibiotics and the dissemination of antibiotic resistance are major concerns in industrial-scale projects that involve bacterial cultures^4^. In particular, metabolic engineering approaches associated with plasmids have several drawbacks including segregational instability, allele segregation, and structural instability^5^. In contrast, integration of relevant genes directly into the chromosome of host strain has many advantages in terms of stability and metabolic burden over the use of vectors. Although recombinant *E. coli* has been used to produce xylitol by some reports^6^,^7^,^8^, XR genes were usually expressed based on plasmid system with inducible promoter. Thus, a multi-copy chromosomal integration of XR genes controlled by constitutive promoter in *E. coli* would be useful for xylitol production.

In industry, xylitol is produced by the reduction of D-xylose derived from hemicellulosic hydrolysate in the presence of Raney nickel catalysts. This process requires the separation of D-xylose from L-arabinose, for that L-arabinose can also be reduced to L-arabitol, an unwanted byproduct^9^. Because D-xylose and L-arabinose are epimers, their separation is very difficult and is one of the main obstacles to the economical production of xylitol. Similarly, in the biotechnological routes, the relaxed sugar specificity of XRs toward L-arabinose is responsible for the formation of L-arabitol^10^. Fortunately, an XR which has an innate 2.4-fold preference for D-xylose over L-arabinose has been isolated from *Neurospora crassa* and a mutant XR (VMCQI) that had a 50-fold lower catalytic efficiency toward L-arabinose was found via several rounds of directed evolution^11^. Furthermore, an engineered *E. coli* (CRP mutant) in conjunction with VMCQI was able to eliminate L-arabitol production from a mixture of D-xylose, L-arabinose, and glucose^7^. However, we used plasmid-based VMCQI in *E. coli*, almost all the L-arabinose in the corncob hemicellulosic hydrolysate has been reduced to L-arabitol in 5L-scale fermentation^2^.

To engineer *E. coli* for more economical production of arabitol-free xylitol from corncob hemicellulosic hydrolysate, the aim of this study is to perform multi-copy chromosomal integration of *N. crassa* XR genes controlled by constitutive promoter in order to achieve stable xylitol production. A plasmid that contained an IS5 sequence (one family of insertion sequences), the R6K ori (narrow-host-range replicon) and the *N. crassa* XR genes driven by a P43 promoter was constructed for chromosomal integration at multiple locations. Moreover, a mutant XR with much lower enzyme activity toward L-arabinose was achieved and a plasmid-free *E. coli* in conjunction with this mutant was constructed. The synergy manifested as increased selectivity such that L-arabitol formation was completely eliminated in xylitol production from corncob hemicellulosic hydrolysate.

## Results and Discussion

### Generating plasmid-free strains for economical production of xylitol

The strong constitutive promoter P43 was isolated from *Bacillus subtilis*^12^ and has been studied extensively. However, little result is known about incorporating P43 in *E. coli* for recombinant protein generation or metabolic engineering. In search of a suitable promoter for more economical production of xylitol, the plasmid pCDF43 with P43 promoter was constructed. After 6 h of cultivation in shake flask, strains HK432 (containing pCDF43) expressed 2758.3 U/L of XR. When cultured in shake flasks, the xylitol productivity of this strain was 0.70 g L^−1^ h^−1^ and this productivity was similar with that of HK402 using the Trc promoter^2^. The results indicated that strain containing the P43 promoter was efficient in producing xylitol. Therefore, P43 promoter was considered as a strong candidate for the production of chemicals especially low value added commodity chemicals in *E. coli*.

Multi-copy integration of the gene of interest into the chromosome is an excellent alternative strategy that overcomes the drawbacks of plasmid-based systems. One system, namely the chemically inducible chromosomal evolution (CIChE) was developed to achieve a plasmid-free, high gene copy expression in *E. coli*^13^. However, strains obtained from these genomic integration carry many antibiotic resistance markers, which might carry a potential risk of spreading these markers to other microbes in nature and add to a rapid emergence of drug-resistant organisms. Therefore, the BICES (biomass-inducible chromosome-based expression system) was established successfully using a temperature-sensitive and counter-selective plasmid in *E. coli* for the production of 2,3-butanediol and acetoin^14^. But, a single gene copy in BICES might be responsible for weak expression. To solve this problem, a similar CIChE method was developed using triclosan-induced chromosomal evolution to integrate more gene copies without incorporating plasmid or antibiotic marker^15^. Yet, it is possible that triclosan resistance gene could transfer horizontally and spread triclosan resistance to wild-type bacteria^16^. Most recently, the CRISPR/Cas9 system has been used in *E. coli* for genome editing^17^, but the gene copy number of chromosomal integration always be single one using CRISPR/Cas9 system in one experiment and large heterologous gene may be not effective using this method^18^.

**Figure 1:**
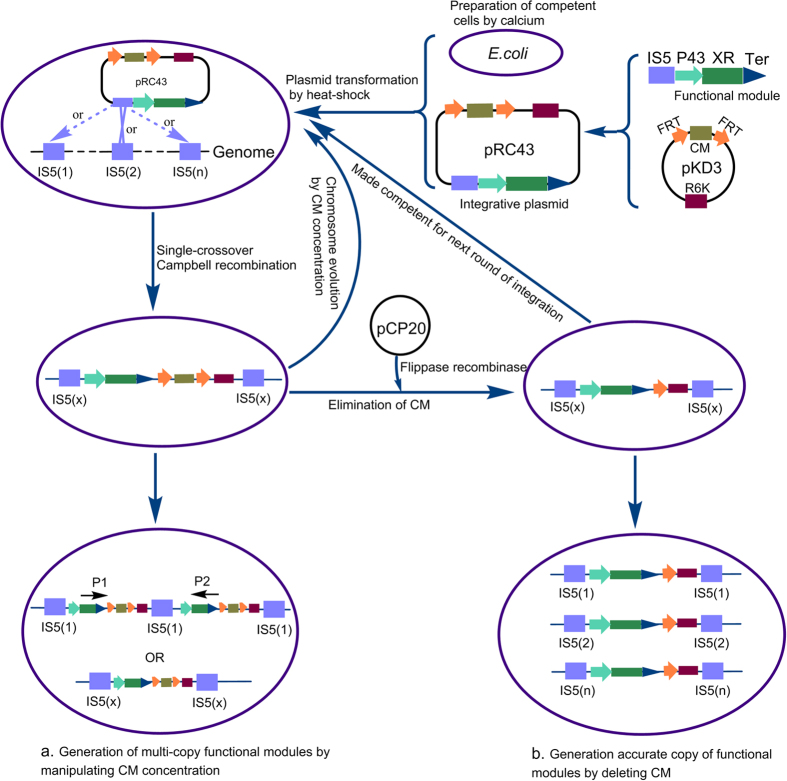
Outline of the RecA-assisted chromosomal integration method in *Escherichia coli.* (**a**) The chromosome will evolve to contain higher gene copy numbers in the presence of chloramphenicol by recA-dependent homologous recombination. (**b**) Accurate copy number can be obtained through a corresponding number of rounds of chromosomal integration by deleting the selectable marker. XR, xylose reductase; IS5, insertion sequence; Ter, terminator; FRT, Flippase recognition target; CM, chloramphenicol; R6K, narrow-host-range replicon; IS5(n), numbers of IS5 sequences; IS5(x), one of the IS5 sequences; P1, IS5-check-P1; P2, IS5-check-P2.

**Figure 2:**
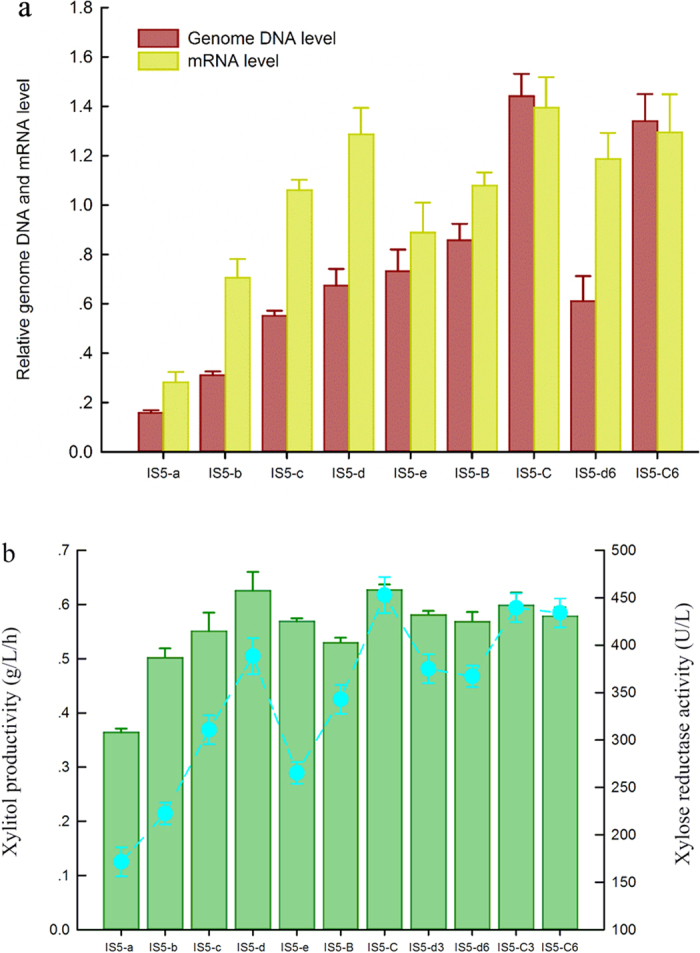
(**a**) Application of RecA-assisted chromosomal integration method to in the generation of plasmid-free *E. coli* strains. The relative genomic DNA and mRNA levels of XR genes were determined by RT-PCR expression analysis. Error bars represent the s.d. (n = 3). (**b**) The xylitol productivity (column) of the plasmid-free strains in the presence of a mixture of glucose and xylose, and the xylose reductase activity (dot) after 6 h of cultivation at 30 °C. Error bars represent the s.d. (n = 3)

Thus, based on RecA-assisted recombination, we described a method for multi-copy chromosomal integration in *E. coli*. Using this method, strains IS5-B and IS5-C were obtained at 400 μg mL^−1^ and 800 μg mL^−1^ chloramphenicol, respectively (Fig. 1a). After five rounds of integrations, five strains IS5-a, IS5-b, IS5-c, IS5-d, IS5-e without antibiotic marker were obtained (Fig. 1b). The mobile insertion element IS5 is a genetically compact DNA sequence of 1195 bp which was found in variable copy numbers in the genome of *E. coli* strains with copy numbers vary from 11 in the sequenced *E. coli* strain MG1655 to 23 in W3110^19^. As integrated loci, insertion sequences were selected on the basis of minimizing unwanted alteration of cellular functions. Our results indicated that the transcription levels of XR genes and the enzyme activity of XR produced by these plasmid-free strains were enhanced after multiple genes integrated into the chromosome, but these parameters were not linearly proportional to the copy numbers of the XR genes (Fig. 2a). In this study, we found that if *N. crassa* XR genes were integrated next to each other, one of the genes could be deleted by the FLP recombinase and resulted in integration failure. Therefore, primers IS5-check-P1 and IS5-check-P2 (Fig. 1a) were used to verify the decentralized assembly strain (*N. crassa* XR genes were distributed randomly in the genome of *E. coli*). In fact, this decentralized assembly strain favors the stability of this strain, since direct repeats of DNA loci are known to be genetically unstable^20^.

### Effect of gene copy number on xylitol production

To investigate the effect of gene copy number on xylitol production, the strains IS5-a, IS5-b, IS5-c, IS5-d, IS5-e, IS5-B, and IS5-C were cultured separately, and shake flask assays were carried out using each strain. The strong constitutive promoter P43 contributed to high xylitol productivity in *E. coli*, but xylitol productivity was not linearly proportional to gene copy number (Fig. 2b). Interestingly, it appeared that the multi-copy genes integration into the chromosome, even at low copy number of XR genes like that of 2 or 4, could effectively lead to sufficient accumulation of xylitol.

In addition to gene integration, the method used in this study allows for the insertion of an exact number of copies of target genes using one vector (may be useful for large genes of interest), which is a feature that is particularly crucial when low gene dosage is needed. In fact, with regard to metabolic engineering in bacteria, low-copy plasmids can perform just as well or better than high-copy plasmids^21^ and two analogous results have been observed in previous studies, in which the highest level of secreted keratinase was achieved in *Bacillus licheniformis* containing between 3 and 5 integrated *kerA* gene, while strain of *Lactococcus lactis* containing more than 6 copies of the *tra904* allele in an intron-GFP cassette exhibited lower levels of keratinase production and reduced GFP expression^22^,^23^. Therefore, methods that provide direct control over gene copy number are especially advantageous for gene expression in many respects.

### Stability of the plasmid-free strains

Potential recombination events at the integration loci during fermentation are of concern given the large number of generations (approximately 120 generations). Strains IS5-d3 (t = 60), IS5-d6 (t = 120), IS5-C3 (t = 60) and IS5-C6 (t = 120) were tested for the stability of the integrated genes. Our results showed that the copy number of the integrated genes and xylitol productivity were not significantly decreased in these strains after many generations (Fig. 2). We therefore concluded that the Campbell-type integrations in these strains were relatively stable. Furthermore, we speculate that this method with multi-copy integration described in this study can be applied to other prokaryotic expression systems to achieve stable strains in high-cell-density fermentation, since insertion sequences including various families are widely distributed in many bacteria^24^.

### High XR total activity has no beneficial effects on xylitol productivity

**Figure 3:**
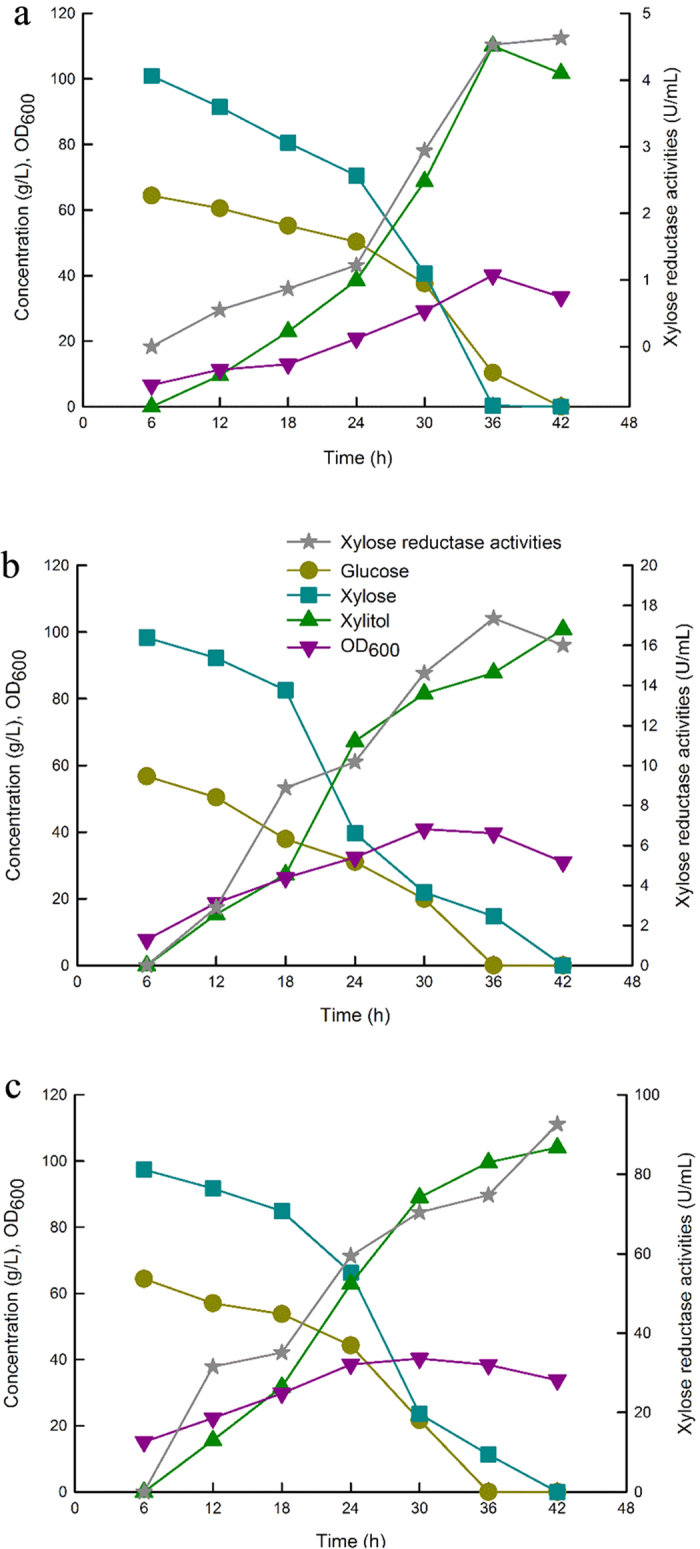
The time-course of batch fermentation of strains IS5-d and HK402 for xylitol production in pure glucose-xylose mixture. (**a**) Batch fermentation of IS5-d in the absence of antibiotic and inducer; (**b**) Batch fermentation of HK402 induced with 0.01 mM IPTG; (**c**) Batch fermentation of HK402 induced with 0.05 mM IPTG.

Strain HK402 was induced with 0.01 and 0.05 mM IPTG respectively, to obtain different levels of XR total activity (highest 17.36 and 92.6 U/mL). Although, the maximum XR total activity of IS5-d (4.63 U/mL) is much lower than that in the plasmid-based system (HK402) with plasmid copy number of about 30, the strain IS5-d produced 110.1 g L^−1^ xylitol at 3.06 g L^−1^ h^−1^ from glucose-xylose mixture in 5 L scale fermentation experiment (Fig. 3a), a productivity that was slightly higher than that containing plasmids (2.4 and 2.48 g L^−1^ h^−1^) (Fig. 3b,c). With regard to the plasmid-free strain (IS5-d) and the plasmid-based system (HK402) induced with different concentrations of IPTG, results from the fermentation experiment indicated that a high concentration of XR in the pathway had no beneficial effects on the production of xylitol. In agreement with our results, strains with almost 2-fold difference of XR total activity had very similar xylitol production, and increasing IPTG concentration during growth resulted in an increase in XR total activity without an increase in xylitol production^25^. Studies suggested that availability of xylose and enzyme do not significantly limit the xylitol production^26^. Therefore, cofactors (NADPH) supply might limit the reduction of xylose to xylitol and would be accounted for our results.

### Low XR total activity is beneficial to decreasing arabitol

**Figure 4:**
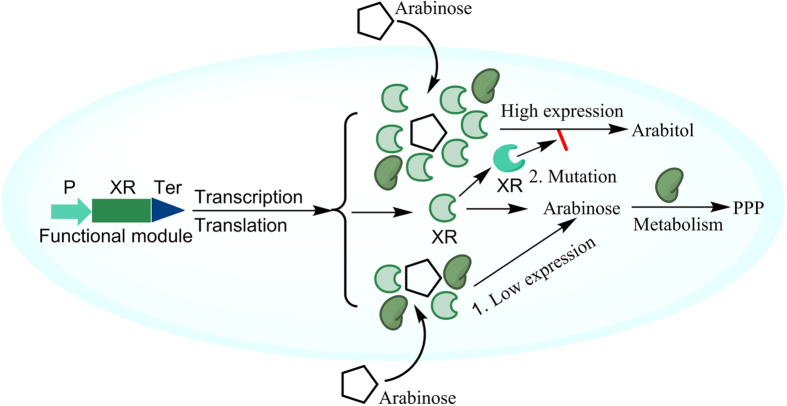
Engineering *Escherichia coli* for arabitol-free xylitol production from corncob hemicellulosic hydrolysate. To minimize the flux from L-arabinose to arabitol, two strategies including: 1) increasing the specificity of XR; 2) decreasing XR total activity can be adopted.

**Figure 5:**
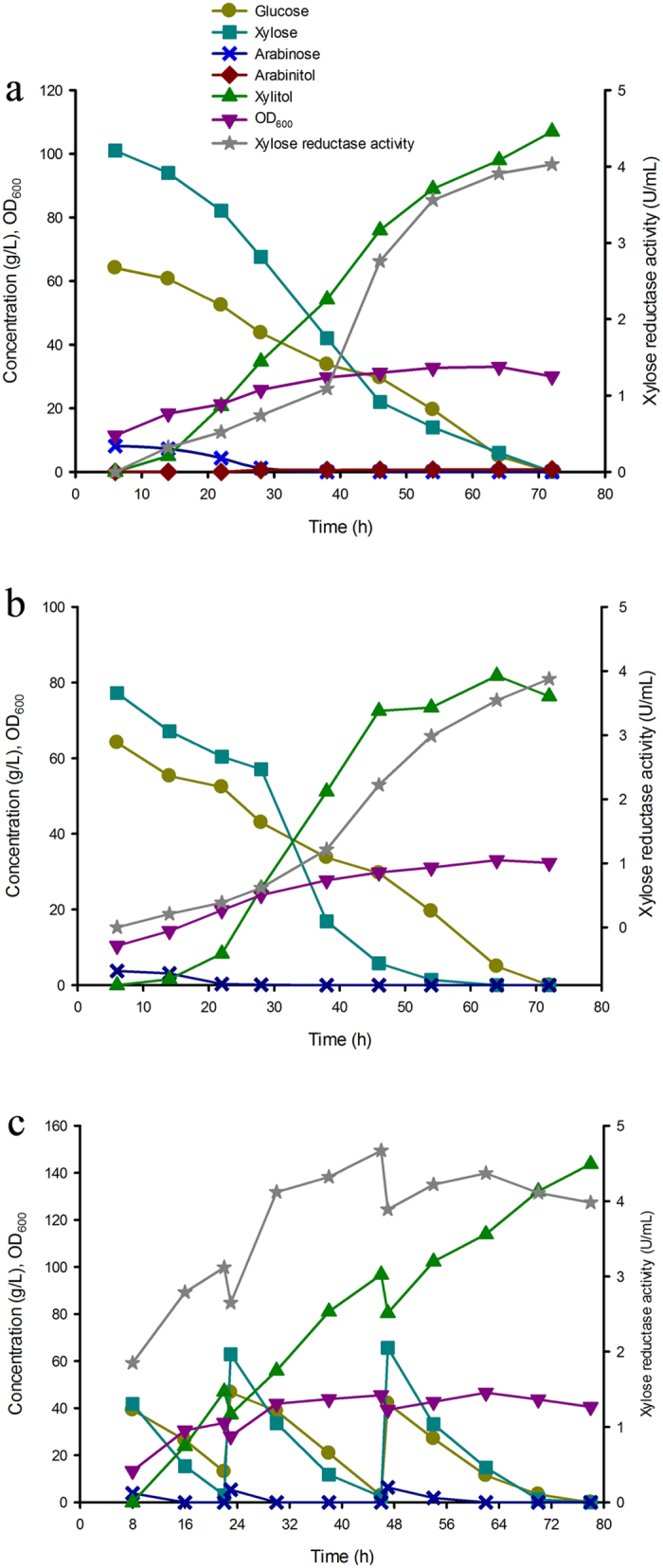
The time-course of batch fermentation by strain IS5-d (**a**) and IS5-M (**b**), and fed-batch fermentation (**c**) by strain IS5-M for xylitol production from corncob hemicellulosic hydrolysate.

Strain HK402 gained high XR total activity, but produced about 5% arabitol^2^. As seen from Table 1, strain HK462 with the eight point-mutations of XR on plasmid pTrc99a can eliminate the formation of arabitol in shake flask fermentation, but still can produce a significant amount of arabitol in the bioreactor fermentation from corncob hemicellulosic hydrolysate. It seemed that more arabinose was metabolized in shake flask fermentation compared with the bioreactor using the same strain, and this indicated that low XR total activity might be beneficial to decreasing arabitol. We speculated that (Fig. 4) arabinose was encompassed by so much XR in the bioreactor, and arabinose was difficult to metabolize by the enzymes responsible for the metabolism of arabinose. Thus, arabinose was reduced to arabitol by XR even with much lower enzyme activity toward L-arabinose. Conversely, arabitol was decreased about 8.8-fold using the plasmid-free strain IS5-d (with much lower XR total activity), and only about 9.6% arabinose was reduced to arabitol (Fig. 5a).

### The selectivity of XR is significantly increased by point mutation

Many efforts have been carried out to switch the cofactor specificity of XR, and varying degrees of success were achieved^27^. Meanwhile, previous work suggested that cofactor induced conformational changes of XR might influence the complimentarity between D-xylose and active site, and cofactor binding could provide an additional screening mechanism of XR for recognizing the substrate more specifically^28^. As the conserved Ile-Pro-Lys-Ser motif was responsible for the cofactor specificity of XR^29^, the point mutation of KSN271-273RTT can influence the complimentarity between XR and cofactor. Thus, we speculate that this mutant might influence the selectivity of XR, and as expected the selectivity of RTT for xylose increased about 29.4% (Table 1). Different mutations always have a synergistic effect on enzyme in protein engineering. Combined mutant RTT with the mutant VMQCI which has a 50-fold lower catalytic efficiency toward L-arabinose evolved by Nair^11^, the mutant VMQCIRTT (eight point-mutations) was obtained by point mutations. The selectivity for xylose of VMQCIRTT was increased by 13-fold and 1.22-fold compared with the WT and VMQCI, respectively. Strain (HK462) containing plasmid with VMQCIRTT produced significantly lower amounts of arabitol than strain HK402, but could not eliminate arabitol formation completely (Table 1).

### Engineering *E. coli* for completely eliminating arabitol in xylitol production from corncob hemicellulosic hydrolysate

In this study, we found that high XR total activity had no beneficial effects on xylitol productivity and low XR total activity was beneficial to decreasing arabitol. So a new strain IS5-M with four copies of VMQCIRTT genes was constructed for xylitol production. In batch fermentation (Fig. 5b), the new strain produced 81.8 g L^−1^ arabitol-free xylitol at 1.27 g L^−1^ h^−1^ and produced 143.8 g L^−1^ arabitol-free xylitol at 1.84 g L^−1^ h^−1^ from corncob hemicellulosic hydrolysate in fed-batch fermentation (Fig. 5c). Although many results about xylitol production from hemicellulosic hydrolysate have been reported, most of the xylitol productivity were too low to be industrial-scale production of xylitol^1^. This might be ascribed to the fact that there were still some toxic components in the detoxified hemicellulose hydrolysate which negatively affected the fermentation performance. Lignocellulose pretreatments always go together with the formation of byproducts that inhibit the fermentation process of *E. coli* and yeast-based systems. It was indicated that detoxification using vacuum evaporation and activated carbon was considered to be an efficient and low-cost procedure to remove the inhibitors for xylitol fermentation^2^. For that the volatile matter such as formic acid, acetic acid and furfural can be detoxified by vacuum evaporation and the non-volatile matter (phenol compounds) can be treated with activated carbon. For more economical production of xylitol, corn steep liquor, glucose from corn and corncob hemicellulosic hydrolysate were used as nitrogen source, co-substrate and substrate, respectively. Thus, this plasmid-free *E. coli* is a candidate for industrial-scale production of xylitol from corncob hemicellulosic hydrolysate.

## Methods

### Strains, plasmids and culture medium

Strains and plasmids used in this study are listed in Table 2. *E. coli* strain DH5α and DH5α λpir (*pir*^+^ for propagating R6K ori plasmids) was used for construction of the recombinant plasmids and HK401was used as the host for gene expression and xylitol production. The gene of *N. crassa* XR (VMCQI) which has five-point mutations (GenBank No.: AY876382.1)^11^,^30^ and various oligonucleotide primers were synthesized by Sangon Biotech (Shanghai, China) (Table 3). *E. coli* strains were cultured in Luria-Bertani (LB) medium (per liter: 10 g tryptone, 5 g yeast extract, and 10 g NaCl) supplemented with 100 μg mL^−1^ ampicillin, 50 μg mL^−1^ kanamycin or 34 μg mL^−1^ chloramphenicol at 37 °C with shake at 200 rpm. For shake flask cultures, modified M9 minimal medium (per liter: 6 g Na_2_HPO_4_; 3 g KH_2_PO_4_; 1 g NH_4_Cl; 0.5 g NaCl) containing MOPS (final concentration: 50 mM, pH 7.4 for pH control) and 5 g L^−1^ of yeast extract were used. For bioreactor studies, modified M9 minimal medium containing 10 g L^−1^ peptone and 7 g L^−1^ yeast extract (or 24 g L^−1^ corn steep liquor) were used.

### Construction of plasmid-free *E. coli*

Plasmid containing the constitutive promoter P43 was constructed based on the vector pCDFDuet-1 using primers P43-P1, P43-P2; P43-XR-P1, P43-XR-P2; pcdf-P1, pcdf-P2; and was designated as pCDF43. For construction of plasmid-free *E. coli*, the plasmid pRC43 containing an IS5 sequence, the R6K ori, a chloramphenicol resistance gene flanked by FRT (Flp recognition) sites and *N. crassa* XR gene under the P43 promoter, was constructed using primers CM + R6K-P1, CM + R6K-P2; IS5-P1, IS5-P2; pCDF43-P1, pCDF43-P2 for repeated *N. crassa* XR genes integrations (Fig. 1). Using this plasmid, we have used two methods for chromosomal integration. One of these (Fig. 1a) is ideal for quick generation and selection of multi-copy functional modules by increasing chloramphenicol concentration, while the other (Fig. 1b) is suited for generating precise copies of functional modules through inducing a corresponding number of rounds of chromosomal integration by deleting the selectable marker.

Briefly, the host strain HK401 was made competent for plasmid transformation using calcium, then the plasmid pRC43 was transformed into HK401 by heat-shock for integration through single-crossover Campbell recombination and the integrants were visually screened on plates containing 34 μg mL^−1^ chloramphenicol. The transformed strain was selected and grown to stationary phase in 34 μg mL^−1^ chloramphenicol. After one round, the integrant was denominated as IS5-a, and was made competent as the start strain for subsequent integration. To efficiently generate multi-copy function modules (Fig. 1a), plasmid pRC43 was transformed into IS5-a by heat-shock, then the culture (about 100 μl) was sub-cultured into a new tube in which the chloramphenicol concentration was increased. The culture was allowed to grow to stationary phase and the process was repeated until the desired concentration was reached. The chromosome was expected to develop higher gene copy numbers by recA-dependent homologous recombination. To obtain accurate number of functional modules (Fig. 1b), plasmid pCP20 was transformed into IS5-a by heat-shock to eliminate chloramphenicol resistance with the aid of FLP recombinase produced from pCP20^31^. Then, the strain (without chloramphenicol resistance) was made competent for next round of integration and the process was repeated until the appropriate gene copy number was achieved.

### Real-time PCR analysis

Total RNA was isolated from strains and purified from genomic DNA using TRIzol reagent (Invitrogen, Carlsbad, CA). The cDNA was prepared from total RNA using AMV reverse transcriptase (Takara, Dalian, China). Quantification of cDNA targets was performed with One Step SYBR^®^ PrimeScript™ RT-PCR Kit II (Takara, Dalian, China). A Bio-Rad CFX96 Real-Time PCR detection system (Bio-Rad, Hercules, CA, USA) was used to measure the expression levels of the target gene. CFX Manager Software (Bio-Rad Laboratories) was used for quantification and 16s RNA gene was used as the internal standard. Gene copy numbers in genomic DNA isolated from the appropriate strains using the Genomic DNA purification kits (Axygen) were measured by the above method. Primers QPCR-F, QPCR-R; 16sRNA-F, 16sRNA-R for Real-time PCR are listed in Table 2.

### Enzyme activity assay

For enzyme activity assay, cultivated cultures were harvested by centrifugation (10,000 × g, 5 min), washed with potassium phosphate buffer (50 mM, pH 7.4) and disrupted by sonication. Cell debris was removed by centrifugation (10,000 × g) for 10 min and the supernatant was used for enzyme activity assays. XR activity was measured by monitoring the oxidation of NADPH (340 nm, 30 °C). The XR assay mixture contained 50 mM MOPS (pH 6.3), 200 μM NADPH, 0.5 M D-xylose, and an appropriate volume of enzyme solution. Enzyme activities were expressed as specific activity [units (mg of protein)^−1^] or total activity (U/L), where one unit of enzyme activity (U) corresponds to the conversion of 1 μM NADPH per minute.

### Shake flask fermentation and the stability assay of plasmid-free strains

All shake flask culture experiments were performed in sterile 250 mL Erlenmeyer flasks containing 45 mL medium. About 1 mL of overnight cultures was inoculated into these flasks and maintained at 37 °C and 200 rpm. After cultures were grown for about 2.5 h to an OD_600_ of about 1.0, 5 mL of a mixture containing 200 g L^−1^ xylose and 100 g L^−1^ glucose were added to the cultures, the flasks were then maintained at 30 °C and 200 rpm.

For the stability assay of plasmid-free strains, overnight *E. coli* cultures were diluted to 10^3^ colony forming units (cfu)/ml in LB and were grown to stationary phase (approximately 20 generations). Six identical transfers in LB medium were carried out to reach 120 generations (t = 120). At t = 0, t = 60 and t = 120, diluted cultures were plated onto LB agar plates and three colonies were randomly selected from each assay time and then cultured in LB. The cultures were subsequently used as seed cultures for xylitol production.

### Effect of XR total activity on xylitol productivity

To investigate the impact of XR total activity on xylitol production, strains HK402 (induced with different concentrations of IPTG to obtain different levels of XR total activity) and IS5-d were used for xylitol production in 5L-scale batch fermentation. The modified M9 minimal medium containing 10 g L^−1^ peptone and 7 g L^−1^ yeast extract was used. The seed culture was inoculated (8% v/v) into the fermenter and the fermentation control settings were: 37 °C, stirring speed at 500 rpm, airflow at 0.8 vvm, and pH at 7.0 (maintained with NH_4_OH). When OD_600_ reached about 15 (about 6 h), a mixture of xylose and glucose with the final concentrations of 100 g L^−1^ and 50 g L^−1^ respectively, supplemented with 20 g peptone, 10 g yeast extract and IPTG (without inducer for IS5-d) were added, then the temperature was decreased to 30 °C. Samples were collected at regular time intervals.

### Using plasmid-free strain for xylitol production from corncob hemicellulosic hydrolysate

The corncob hemicellulosic hydrolysate (prepared using dilute acid) which was provided by Huakang Pharmaceutical Co. (Quzhou, Zhejiang province, China) was prepared by the method described in our previous work^2^ and was concentrated under vacuum to achieve appropriate concentration of xylose. Batch xylitol production from corncob hemicellulosic hydrolysate by IS5-d was carried out in a 5 L-scale laboratory fermenter containing 3 L modified M9 minimal medium supplemented with 24 g L^−1^ corn steep liquor and the fermentation control settings were described above. When OD_600_ reached about 15, corncob hemicellulosic hydrolysate supplemented with appropriate amount of glucose (for cell growth and NADPH supply) and 30 g corn steep liquor were added.

### Increasing the selectivity of XR by point mutation

According to the previous results^27^, the point mutation (KSN271-273RTT) which can increase the activity of XR was carried out based on XR (VMCQI) by the Megaprimer PCR of Whole Plasmid method^32^ using primers poit-M1-F and poit-M1-R. Thus, the VMCQIRTT (eight point-mutations) mutant was obtained and verified by DNA sequencing. The point mutation of the integrative plasmid pRC43 was also implemented using the primers poit-M1-F and poit-M1-R and the mutant plasmid was designated as pRC43M. The wild type of XR (WT) was obtained from XR (VMCQI) by point mutation using primers poit-M2-F and poit-M2-R, and the three point-mutations (RTT) was achieved from XR (WT) using primers poit-M1-F and poit-M1-R.

### Engineering plasmid-free *E. coli* for arabitol-free xylitol production

The plasmid-free *E. coli* IS5-M with four copies of VMCQIRTT was obtained by the same method described above. Batch fermentation for IS5-M was performed according to the method above. Fed-batch fermentation was accomplished in a 15 L-scale bioreactor initially containing 6 L modified M9 minimal medium containing 24 g L^−1^ corn steep liquor. The culture conditions such as agitation speed, inoculum size, pH, and aeration rate, were the same as those of batch fermentation except that the temperature was controlled at 30 °C all the way. The agitation speed was controlled to maintain the dissolved oxygen (D.O.) at about 20%. When OD_600_ reached about 15 (about 8 h), concentrated corncob hemicellulosic hydrolysate (the final concentration of about 50 g L^−1^ xylose) supplemented with appropriate amount of glucose and 20 g L^−1^ (final concentration) corn steep liquor were added. At 22 and 46 h, 2 L of concentrated corncob hemicellulosic hydrolysate (the final concentrations of about 60 g L^−1^ xylose) supplemented with an appropriate amount of glucose and 15 g L^−1^ corn steep liquor were added to the bioreactor all at once. The total xylose concentration in the corncob hemicellulosic hydrolysate for fed-batch fermentation was about 150 g L^−1^ and the overall fermentation volume was 12 L. At regular time intervals, samples were acquired for analysis.

### Analyses

Xylitol, arabitol, glucose, arabinose and xylose were quantified using a Dionex UltiMate 3000 ultra-high performance liquid chromatography (UHPLC) system equipped with a Corona Charged Aerosol Detector (CAD). The analytical column was an Φ 7.8 mm × 300 mm Aminex HPX-87C column (Bio-Rad Laboratories). Pure water was used as mobile phase (0.6 mL min^−1^, 76 °C).

## Additional Information

**How to cite this article**: Su, B. *et al.* Construction of plasmid-free *Escherichia coli* for the production of arabitol-free xylitol from corncob hemicellulosic hydrolysate. *Sci. Rep.* **6**, 26567; doi: 10.1038/srep26567 (2016).

## Figures and Tables

**Table 1:** The study of mutant XRs in shake flask fermentation except where otherwise noted.

Mutant	XR activity for xylose^a^	XR activity for arabinose^a^	Selectivity^b^	Total XR activity^c^	Arabitol/Arabinose^d^ (%)	Xylitol productivity (g L^−1^h^−1^)
HK442 (WT)	18.97 ± 3.86	22.44 ± 4.32	0.85	4.09 ± 0.17	81.96 ± 8.96	0.46 ± 0.07
HK452 (RTT)	31.62 ± 5.33	28.76 ± 4.78	1.10	5.34 ± 0.35	76.28 ± 4.56	0.47 ± 0.12
HK402 (XR)	16.32 ± 2.98	3.06 ± 1.21	5.33	3.96 ± 0.31	18.25 ± 2.35	0.51 ± 0.09
HK462 (VMCQIRTT)	9.6 ± 1.67	0.81 ± 0.11	11.85	3.55 ± 0.22	0	0.43 ± 0.15
HK402^e^	–	–	–	38.60	93.75	1.4
HK462^e^	–	–	–	22.57	47.34	1.29

^a^XR activity (U/mg pro) was determined using xylose and arabinose as substrate, respectively.

^b^Selectivity = XR activity for xylose/XR activity for arabinose.

^c^The highest XR activity (U/mL) in the fermentation using xylose as substrate.

^d^Arabitol/Arabinose = arabitol in the production/arabinose in the hemicellulosic hydrolysate.

^e^The experiments were carried out in 5 L-scale bioreactor.

**Table 2:** *Escherichia coli* strains and plasmids used in this study.

Strain/plasmid	Description	Source
Strains		
DH5α	*supE44* Δ*lacU169 (φ80 lacZ*Δ*M15) hsdR17 recA1 endA1 gyrA96 thi-1 relA1*	Invitrogen
HK401	*E. coli* W3110, Δ*ptsG::FRT*, Δ*xylAB::FRT*, Δ*ptsF::FRT*	Su *et al.*^2^
HK402	HK401 with pTrc99a-rbs-xr6600	Su *et al.*^2^
HK432	HK401 with pCDF43	This study
HK442	HK401 with pTrc99a-xr-WT	This study
HK452	HK401 with pTrc99a-xr-3	This study
HK462	HK401 with pTrc99a-xr-8	This study
IS5-a	HK401 with one round of integration using pRC43	This study
IS5-b	HK401 with two rounds of integration using pRC43	This study
IS5-c	HK401 with three rounds of integration using pRC43	This study
IS5-d	HK401 with four rounds of integration using pRC43	This study
IS5-e	HK401 with five rounds of integration using pRC43	This study
IS5-B	IS5-a integration with 400 μg mL^−1^ chloramphenicol	This study
IS5-C	IS5-B integration with 800 μg mL^−1^ chloramphenicol	This study
IS5-C60	IS5-C subculture for approximately 60 generations	This study
IS5-C120	IS5-C subculture for approximately 120 generations	This study
IS5-d60	IS5-d subculture for approximately 60 generations	This study
IS5-d120	IS5-d subculture for approximately 120 generations	This study
IS5-M	HK401 with four rounds of integration using pRC43M	This study
Plasmids^a^		
pCDFDuet-1	pCloDF13-derived vector; T7 promoter, Str^R^	Novagen
pTrc99a-rbs-xr6600	pTrc99a with XR and RBS from pET-30a(+)	Su *et al.*^2^
pTrc99a-xr-WT	pTrc99a with the wild type XR and RBS from pET-30a(+)	This study
pTrc99a-xr-3	pTrc99a with three point mutations XR and RBS from pET-30a(+)	This study
pTrc99a-xr-8	pTrc99a with eight point mutations XR and RBS from pET-30a(+)	This study
pCDF43	XR under the P43 promoter	This study
pRC43	XR under the P43 promoter, R6K ori, including the IS5 sequence	This study
pRC43M	eight point mutation XR under the P43 promoter, R6K ori, include the IS5 sequence	This study
pKD3	Template plasmid with cat gene and FLP recognition target	Datsenko and Wanner^31^
pCP20	FLP recombinase helper plasmid	Datsenko and Wanner^31^

^a^Amp^R^, ampicillin; Kan^R^, kanamycin; Str^R^, Streptomycin; R, resistance.

**Table 3:** Primers used in this study.

Primer	Sequence (5′-3′)	Restriction enzyme
P43-P1	CCGGAATTCGAGCTCAGCTTTATTGAGTGGATGA	*Eco*RІ
P43-P2	GTTGAGTTTGATCGCAGGTACCATTTGTTTTCCTCCTTGTTCCGT	
P43-XR-P1	ACGGAACAAGGAGGAAAACAAATGGTACCTGCGATCAAACTCAAC	
P43-XR-P2	CCCAAGCTTCTAACCGAAAATCCAGAGGTTCTC	*Hin*dІІІ
pcdf-P1	CCCAAGCTTCTGCTGCCACCGCTGAGCAATAACTAGCATAACCCCTT	*Hin*dІІІ
pcdf-P2	CCGGAATTCGCGGTTCAGTAGAAAAGATCAAAGGATC	*Eco*RІ
CM + R6K-P1	AAAACTGCAGAGTAGGGAACTGCCAGGCATCAA	*Pst*І
CM + R6K-P2	AGTGGGAGAGATCTCACTAAGGTGCCTCACTGATTAAGCATTGG	
IS5-P1	CCGGAATTCAAGAGATTTTCTTGTCCCGCATG	*Eco*RІ
IS5-P2	TGCTTAATCAGTGAGGCACCTTAGTGAGATCTCTCCCACTGACGTAT	
pCDF43-P1	CCGGAATTCGAGCTCAGCTTTATTGAGT	*Eco*RІ
pCDF43-P2	AAAACTGCAGTGCTGGTTTACCGGTTTATTGACTA	*Pst*І
IS5-check-P1	CACTTTATATTTTACATAATCGCGCG	
IS5-check-P2	TCCTATACTTTCTAGAGAATAGGAACTTCG	
QPCR-F	GACGGCAAGAGCGAGAT	
QPCR-R	TGCTGGACGAGGTAGGG	
16sRNA-F	ACCCTTATCCTTTGTTGCC	
16sRNA-R	TATGAGGTCCGCTTGCTCT	
poit-M1-F	CATCATCCCCCGCACTACCCGCGAGGCCACCATGA	
poit-M1-R	TCATGGTGGCCTCGCGGGTAGTGCGGGGGATGATG	
poit-M2-F	ACTGGGGTCTCGAGTACTTCGATCTCTACCTGATCCACTTCCCCGTCGCCCT	
poit-M2-R	AGGGCGACGGGGAAGTGGATCAGGTAGAGATCGAAGTACTCGAGACCCCAGT	
